# Dairy Consumption and Its Impact on PCOS and the Reproductive System: The Connection

**DOI:** 10.7759/cureus.82116

**Published:** 2025-04-11

**Authors:** Fatimah M Ahmad, Ariel Benor

**Affiliations:** 1 Obstetrics and Gynecology, American University of Antigua, Antigua and Barbuda, USA; 2 Obstetrics and Gynecology, West Virginia University Berkeley Medical Center, Martinsburg, USA

**Keywords:** cow’s milk, endocrine pathology, female reproductive health, polycystic ovary syndrome (pcos), resistance to insulin

## Abstract

Polycystic ovary syndrome (PCOS) is a prevalent and complex endocrine disorder that affects many women of reproductive age. It is characterized by hyperandrogenism, ovulatory dysfunction, and polycystic ovarian morphology. PCOS is associated with an increased risk of cardiovascular disease, obesity, diabetes, and other long-term health conditions, including cancer. Given its widespread impact, it should be recognized as a significant public health concern, highlighting the urgent need to investigate its underlying causes and the behavioral factors contributing to its rising prevalence. The increasing prevalence of PCOS is closely linked to the global and national rise in obesity. Alarmingly, a significant portion of cases remain undiagnosed. Although the etiology of PCOS has yet to be elucidated, the general consensus is that obesity and insulin resistance (IR) are likely strong contributing factors. Although the etiology of IR is multifactorial, some believe that it may be associated with dairy consumption. Dairy, particularly cow’s milk, has been a staple in the Western diet for decades; however, over the past 50 years, due to the popularization from marketing campaigns promoting it as beneficial for bone health, its consumption has now skyrocketed. There has been a growing focus on the role of dairy products on disease, especially regarding their impact on ovulation, fertility, and endocrinologic/metabolism disorders. Here, we attempt to review the contemporary evidence examining the possible role and relationship of dairy products to the pathophysiology of PCOS. We hope to clarify to the reader, based on the best available evidence, whether a low-dairy diet may help improve PCOS parameters and its comorbid conditions. This review aims to explore this question with the goal of addressing gaps in the current understanding of the interplay between dairy consumption and hormonal/metabolic dysfunction.

## Introduction and background

Polycystic ovary syndrome (PCOS) is a widely common endocrinopathy that affects up to 18% of women of reproductive age [1], manifested by menstrual irregularity, infertility, and hyperandrogenic symptoms. It is estimated that around 90-95% of ovulatory infertility cases are caused by PCOS [2]. Its growing prevalence is closely tied to the rise in obesity, both in the United States and around the world [3,4]. Despite its serious impact, as many as 70% of PCOS cases go undiagnosed, leaving many women unaware of the condition and its risks [3,4]. It presents itself with a spectrum of symptoms, most notably in patients suffering from infertility, complications of pregnancy, and various cardiometabolic conditions, including insulin resistance (IR), hyperinsulinemia, dyslipidemia, and the accumulation of visceral fat [1]. Additionally, patients with PCOS may face psychosocial challenges, including depression, anxiety, and eating disorders, which are comorbid conditions [1]. The CDC states that in the United States, PCOS affects around 5-6 million women [5]; hence, research into the impact of environmental and nutritional factors is vital to the well-being of a large cohort of women. The complex etiology of PCOS is multifactorial, involving genetic, environmental, and behavioral factors. Among these, diet has emerged as a significant area of interest, particularly the role of dairy consumption [6,7].

While carbohydrates are known to stimulate insulin secretion, studies indicate that dairy products and starches trigger a greater postprandial insulin response compared to non-starchy vegetables and fruits [8]. Cow milk and dairy products are dietary staples in many Western societies, where their consumption is widely endorsed for their benefits in calcium absorption, bone health, and protein intake. Despite these benefits, the long-term negative effects of milk and milk proteins on health have often been overlooked. Recent hypotheses suggest that milk protein consumption could be a significant environmental factor contributing to various chronic diseases prevalent in Western societies. Specifically, milk protein may lead to postprandial hyperinsulinemia and alter the growth hormone/insulin-like growth factor-1 (IGF-1) axis, resulting in persistently elevated IGF-1 levels. The insulin/IGF-1 signaling is implicated in regulating fetal growth, T-cell maturation, linear growth, and the development of conditions such as acne, atherosclerosis, diabetes mellitus, obesity, and cancer [9]. In addition, epidemiological evidence suggests that increased milk consumption correlates with higher BMI and earlier onset of puberty [9], both risk factors for metabolic disorders such as IR, PCOS, and type 2 diabetes mellitus (T2DM).

Some studies suggest that markers like TNF-α (a pro-inflammatory cytokine) were higher in the population consuming whole milk, contributing to the development of these chronic comorbidities [10]. In addition, milk proteins contain exosomal microRNAs, such as oncogenic microRNA-21 and let-7, which may influence cancer progression and IR [9]. González et al., examining the effects of high saturated fat intake, such as those in milk, on inflammatory pathways in women with PCOS and healthy controls, demonstrated that consuming saturated fat led to an increase in NF-κB activation, a key factor in inflammation, which persisted for at least three hours [11]. This was accompanied by higher levels of TNF-α and C-reactive protein. Notably, the inflammatory response was not driven solely by obesity, as obesity amplified but did not initiate the response. The inflammatory markers were also correlated with IR, dyslipidemia, and androgen levels, suggesting that nutrient-induced inflammation may trigger these metabolic and hormonal changes in PCOS.

Obesity is known to be one of the greatest risk factors of PCOS, with visceral obesity in PCOS patients being linked to increased IR, which contributes to the development of reproductive disorders [12]. Koseci et al. explored the impact of IR on PCOS by examining the relationship between NPY and IR [13]. The neuropeptide Y (NPY) system is believed to play a crucial role in regulating energy balance and contributing to the pathophysiology of obesity [14]. NPY, which enhances appetite and is structurally similar to pancreatic polypeptide, plays a role in controlling nutrient intake and body weight, and it inhibits the hypothalamo-hypophyseal-ovarian (HPO) axis [13]. NPY has also been identified as a key regulator of ovarian granulosa cell function, and findings have demonstrated that NPY stimulates the proliferation of granulosa cells in preantral and early antral follicles while promoting apoptosis in granulosa cells of late antral follicles [15,16]. The study involved 45 women with PCOS and 44 healthy women of reproductive age. During the early follicular phase, various hormone levels, including insulin, fasting blood sugar, follicle-stimulating hormone (FSH), luteinizing hormone (LH), and NPY, were measured. The findings revealed that obese women with PCOS had higher fasting insulin levels and homeostatic model assessment for IR (HOMA-IR) compared to normal-weight individuals and healthy controls. HOMA-IR is currently considered the best and most reliable marker; however, a universally accepted cut-off point for diagnosing IR in PCOS has yet to be established [17]. They concluded that although NPY levels were elevated in obese women with PCOS, this difference was not statistically significant, and NPY levels showed no variation between those with and without IR [13]. A rat model of PCOS with chronic androgen exposure demonstrated that NPY inhibits androgen-induced apoptosis in granulosa cells, which suggests that NPY present in follicular fluid may play a role in the pathogenesis of follicular developmental failure in PCOS [18]. In addition, elevated insulin levels are thought to contribute to or exacerbate comorbidities and lead to higher androgen levels in women with PCOS [19,20]. Baldani et al. [21] examined the effects of obesity on PCOS and included a total of 74 obese and 208 non-obese females with PCOS. Obese females with PCOS were found to have higher rates of developing oligomenorrhea, hyperandrogenemia, IR, hypercholesterolemia, hypertriglyceridemia, and inflammation (marked by CRP) than non-obese females [21]. Among obese females with PCOS, metabolic dysfunction was much more likely to develop than in their non-obese counterparts [21]. However, obesity is independently linked to IR [22] and imbalances in sex steroids, which can heighten the likelihood of menstrual irregularities and hyperandrogenism, manifested by symptoms of PCOS [23].

Lawlor et al. [24] aimed to explore the link between milk consumption and metabolic health in women. Out of 4,024 women surveyed, 111 (2.8%) reported never drinking milk. These women generally showed lower IR (HOMA scores), lower triglycerides, lower BMI, and higher HDL levels, as well as lower odds of metabolic syndrome compared to those who drank milk. The study’s findings are consistent with other research, showing that individuals who avoid milk or are lactose intolerant tend to have lower fasting glucose levels and improved lipid profiles [24].

Therefore, one of the goals of the medical community should be to identify and evaluate the behavioral factors that may be contributing to PCOS not only in order to ameliorate the burden on the healthcare system as a whole but also to proactively decrease the long-term health risks in fetal programming that can arise from disruption in the insulin-IGF-1 signaling.

## Review

Methodology

Research Strategy

We conducted a search across multiple databases, including ScienceDirect, DeepDyve, and PubMed, using specific keywords such as “PCOS,” “milk consumption,” “insulin resistance,” and “endocrine imbalance.” To ensure relevance, we prioritized studies from the past 10 years, selecting articles published after 2000 for further analysis. However, we did not exclude studies published before 2000 if they provided valuable insights.

Inclusion Criteria

Articles that were case reports, case series, cross-sectional studies, review articles, commentaries articles published more than five years ago, and cohort or controlled trials that included women were included. For this review, we carefully chose studies that reported a significant finding in endocrine abnormalities relative to milk or dairy consumption in the female population.

Exclusion Criteria

Articles that had findings in the male population were excluded as they were not relevant to the main topic, which is a pathology seen in the female population. In addition, findings that reported associations to other pathologies such as cancers were excluded.

Results

**Figure 1:**
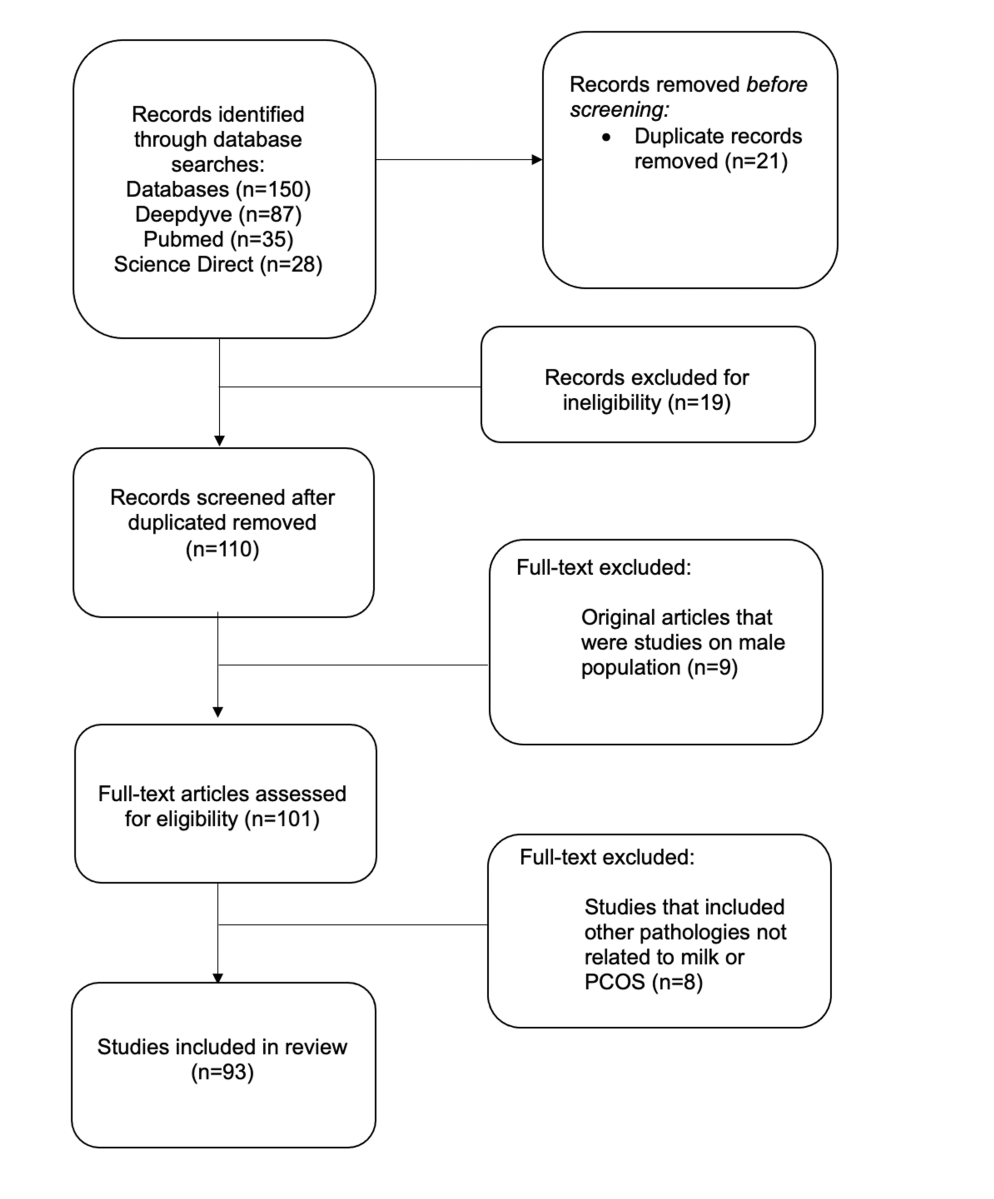
Flow diagram depicting the selection process of the included articles used in this review

We conducted a data search that identified 150 articles. After eliminating duplicates, 129 articles remained and were screened based on their titles and abstracts. Articles that did not meet the eligibility criteria were excluded. A further review was performed by examining the full text of the articles, focusing on the discussion and conclusion sections to exclude studies that did not provide the necessary data for this review, such as those involving male populations, unrelated endocrine conditions, or cancers. This process led to the inclusion of 93 articles relevant to our objective (Figure 1).

Low-fat vs. high-fat dairy consumption 

Some studies have suggested that low-fat dairy consumption may have beneficial effects on our health by reducing the risk of developing IR and T2DM. Milk and dairy are highlighted as rich sources of nutrients such as calcium, vitamin D, magnesium, and probiotics, all of which are potentially beneficial for metabolic health [2]. Despite these benefits, there are conflicting findings regarding the impact of dairy consumption on insulin sensitivity (SI) and resistance in women. Some studies suggest that dairy consumption is typically inversely related to the risk of T2DM, especially concerning low-fat and fermented dairy products [25]. For instance, an increase of 200 g in daily low-fat dairy intake was linked to a 4% decrease in the risk of developing T2DM, whereas full-fat dairy did not demonstrate the same benefit [25]. In addition, it was determined that women from lower socioeconomic backgrounds, smokers, and those with less healthy diets were more likely to consume full-fat milk [24]. Several studies highlight that regular intake of dairy, particularly low-fat varieties, may improve SI and reduce the risk of IR, while others indicate a neutral or even negative association, especially in women with higher dairy consumption. Findings from studies [3,26,27] emphasize the health benefits of low-fat dairy products, including skim milk and yogurt, with consistent risk reduction observed in additional research [28,29]. Additionally, yogurt stands out as particularly beneficial due to its association with increased concentrations of anorexic peptides, glucagon-like peptide-1 and peptide YY (GLP-1 and PYY), which improve glucose homeostasis. Studies show that regular consumption of yogurt significantly reduces the risk of T2DM in women [30,31]. This finding is supported by a study that found that women consuming over 2.9 servings of dairy per day had a 20% lower risk compared to those who consumed less [2]. Fermented dairy products rich in probiotics, such as yogurt, are particularly advantageous as they aid in glucose metabolism and may decrease the risk of T2DM [2]. Overall, while dairy products, especially low-fat and fermented types, appear to provide protective benefits against T2DM in women, the effects of full-fat dairy are less clear [2]. Chen et al. [32] demonstrate that full-fat yogurt notably reduced HOMA-IR, fasting insulin levels, and two-hour glucose levels following a 75 g oral glucose tolerance test, compared to full-fat milk, in women with metabolic syndrome and non-alcoholic fatty liver disease. Interestingly, while milk consumption also significantly lowered HOMA-IR, fasting glucose, and insulin levels, it led to a marked increase in insulin levels during the two-hour oral glucose tolerance test. The authors proposed that the adverse effects of milk on carbohydrate metabolism were not linked to reduced SI but rather to milk’s potential to prolong postprandial insulin secretion [32].

Conversely, some studies have suggested that even though a reduced or low-fat dairy diet may be beneficial in reducing BMI, hypertension, and IR, it may be associated with higher triglycerides. A cross-sectional study in Ireland [10] was conducted, and the study involved 1500 healthy Irish adults (aged 18-90 years) who completed a four-day food diary to record dairy consumption (milk, cheese, yogurt, cream, and butter). Fasting blood samples (from 897 participants) and anthropometric measurements were also collected. Dietary patterns were analyzed using cluster analysis based on dairy consumption. Cluster analysis identified three dairy consumption patterns: ‘Whole milk,’ ‘Reduced fat milks and yogurt,’ and ‘Butter and cream.’ Results displayed that the ‘Reduced fat milks and yogurt’ pattern scored highest on a Healthy Eating Index but showed higher triglyceride and total cholesterol levels. In addition, higher cheese consumption was linked to higher C-peptide levels. Furthermore, Chavarro et al. [33] suggest that high intakes of yogurt are associated with an increased risk of anovulatory infertility.

In regard to the effect of dairy on reproduction, some studies reveal a neutral or inverse association between total dairy intake and ovulatory infertility. For instance, increasing the intake of low-fat milk products by one serving per day was associated with an 11% rise in the risk of ovulatory infertility, whereas adding a serving of whole milk without increasing overall energy intake was linked to a reduction in risk by more than 50% [33]. The authors attributed this difference to the higher estrogen content in high-fat dairy products, which resulted in a more moderate increase in serum IGF-1 levels compared to low-fat options [33]. Additionally, they suggested that the connection between dairy consumption (both low-fat and full-fat) and infertility due to anovulation was more pronounced in women who did not exhibit certain clinical signs of PCOS than in those who did [33].

The role of diet modification in managing PCOS comorbidities

While there is still limited research on dairy consumption and its relation to PCOS, there has been a significant association, specific to cow’s milk, and PCOS. A study conducted at Shahid Beheshti Hospital in Isfahan University of Medical Science, Isfahan, Iran, utilized a descriptive cross-sectional design involving 400 women. Dietary intake was assessed using a validated food frequency questionnaire, alongside other variables such as genetic predisposition, ovarian disease history, age at menarche, and physical activity levels. The key findings indicate a significant direct relationship between milk consumption and PCOS risk (p = 0.028), suggesting that higher milk intake may increase the likelihood of developing PCOS. However, no significant associations were observed between PCOS and other dairy products like yogurt, sherbet/frozen yogurt, cheese, and cream [34].

A study from 2015 [8] conducted a prospective eight-week low starch/low dairy diet intervention in women with PCOS. The study included 24 overweight and obese women (BMI ≥25 kg/m² and ≤45 kg/m²) diagnosed with PCOS, who followed the diet without calorie counting or medication changes. Weight, BMI, weight circumference, waist-to-height ratio, fasting and two-hour glucose and insulin, HOMA-IR, HbA1c, and total and free testosterone were measured before and after the eight-week intervention. The results displayed significant improvements across several measures: the participants lost an average of 8.6 kg in weight, had a reduction of waist circumference (−8.4 ± 3.1 cm, p < 0.001), and improved SI measured by fasting insulin (−17.0 ± 13.6 μg/mL, p < 0.001) and HOMA-IR (−1.9 ± 1.2, p < 0.001). Additionally, decreased levels of total testosterone (−10.0 ± 17.0 ng/dL, p = 0.008) and free testosterone (−1.8 pg/dL, p = 0.043) were determined, along with decreased scores (Ferriman-Gallwey) assessing hirsutism severity (−2.1 ± 2.7 points, p = 0.001). This study concluded that reducing insulinemic foods, i.e., high starch/high dairy content, may offer a promising approach to managing PCOS symptoms, including weight gain and hormonal imbalances.

Metabolic inflexibility, or the inability to transition between lipid oxidation and glucose oxidation in response to insulin stimulation, has been identified as a key characteristic in women with PCOS. This condition is likely linked to IR and compensatory hyperinsulinemia [35]. Another study by Pohlmeier et al. [36] was conducted to determine whether a low-starch/low-dairy diet would improve fasting and postprandial fat oxidation after a high saturated fat liquid meal (HSFLM) in overweight and obese women. Indirect calorimetry was employed both during fasting and for five hours following the consumption of HSFLM to assess the respiratory exchange ratio (RER), macronutrient oxidation, and energy expenditure (EE) at both week 0 and week 8. The diet excluded high insulinemic foods like grains, dairy, and sugars, promoting the consumption of lean proteins, non-starchy vegetables, fruits, nuts, and oils. Measurements of these parameters are shown in Table 1.

**Table 1:** Results of low-starch/low-dairy diet in improving fasting and postprandial fat oxidation after an HSFLM in overweight and obese women at both weeks 0 and 8 HSFLM, high saturated fat liquid meal; RER, respiratory exchange ratio; EE, energy expenditure; CHO, carbohydrate

RER	EE	Fat oxidation	CHO oxidation
Fasting RER significantly decreased from 0.85 ± 0.05 to 0.77 ± 0.02 after the diet intervention (p < 0.001). Postprandial RER showed no significant change overall, indicating that the major effect of the diet was on fasting RER rather than postprandial responses.	Adjusted AUC EE decreased significantly from 114.5 ± 13.1 to 105.7 ± 9.8 kcal (p = 0.005) after the diet, primarily due to weight loss rather than diet alone. There was no significant change in EE/kg after adjusting for body weight.	Fasting and postprandial fat oxidation increased significantly after the diet intervention (p < 0.001), indicating improved metabolic flexibility in utilizing fats.	Fasting and postprandial CHO oxidation decreased significantly after the diet intervention (p < 0.001), reflecting a reduced reliance on carbohydrates for energy.
No significant correlations were found between changes in fasting insulin levels and changes in RER after adjusting for weight, BMI, and hormone levels.	The diet did not affect EE independently; rather, the decrease was attributed to the accompanying weight loss.	Significant increases were observed in AUC fat oxidation values, suggesting enhanced fat metabolism throughout the day.	AUC CHO values also decreased significantly, indicating overall reduced carbohydrate utilization over time.

Results showed significant improvements in several measures post-intervention: participants experienced reductions in body weight, BMI, waist circumference, and fat mass. Fasting insulin levels significantly decreased, and there was a notable improvement in fasting and postprandial fat oxidation rates as measured by indirect calorimetry. However, other parameters, such as glucose levels and free testosterone, did not show significant changes.

According to Rosenberg [37], obese individuals have exacerbated IR, driven by excess intra-abdominal fat that releases free fatty acids and various cytokines, leading to metabolic dysfunction. This results in lipotoxicity, where fat accumulates in non-adipose tissues, further hindering insulin action [37]. Notably, hepatic IR is present in obese women with PCOS, but it is absent in healthy women of comparable weight, highlighting a distinct metabolic challenge associated with PCOS [37].

The impact of milk protein on reproductive pathophysiology

**Figure 2:**
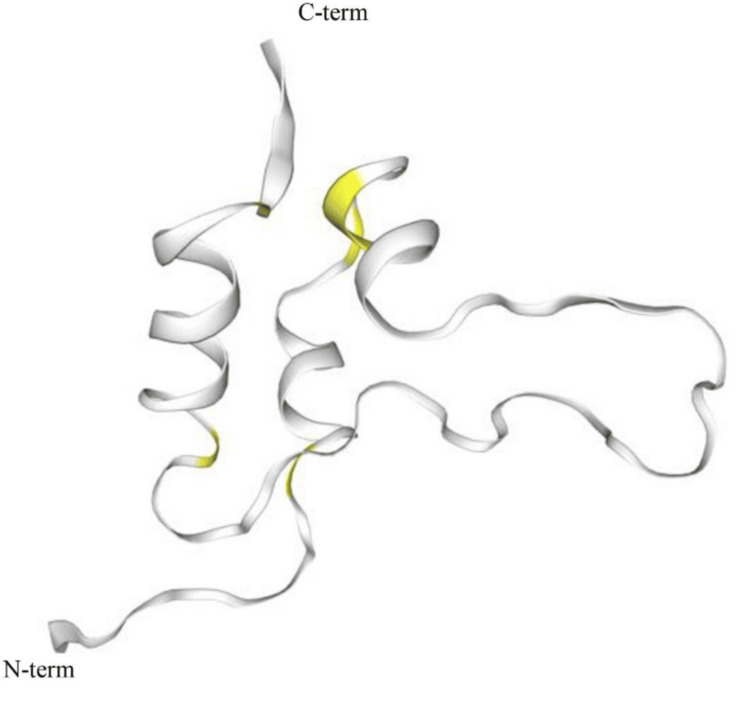
Bovine IGF-1 protein From reference [41]. Copyright © 2025 Elsevier B.V. Reprinted with permission. IGF-1, insulin-like growth factor 1

Milk serves as a vital source of calcium and supports bone growth and mineralization, which are positively correlated with serum IGF-1 levels [38]. Cow’s milk contains significant amounts of bioactive hormones, including IGF-1 and IGF-2 [39]. IGF-1 plays a crucial role in lactogenesis. When cows are treated with recombinant growth hormone to boost milk production, their milk contains elevated levels of IGF-1, and additionally, elevated IGF-1 levels remain present even after milk undergoes pasteurization and homogenization [40]. Interestingly, bovine IGF-1 and human IGF-1, as shown in Figure 1 and Figure 2, respectively, have an identical amino acid sequence, allowing them to bind to the human IGF-1 receptor [40]. Evidence suggests that IGFs present in milk can resist digestion and remain biologically active in the plasma of those who consume milk [40].

**Figure 3:**
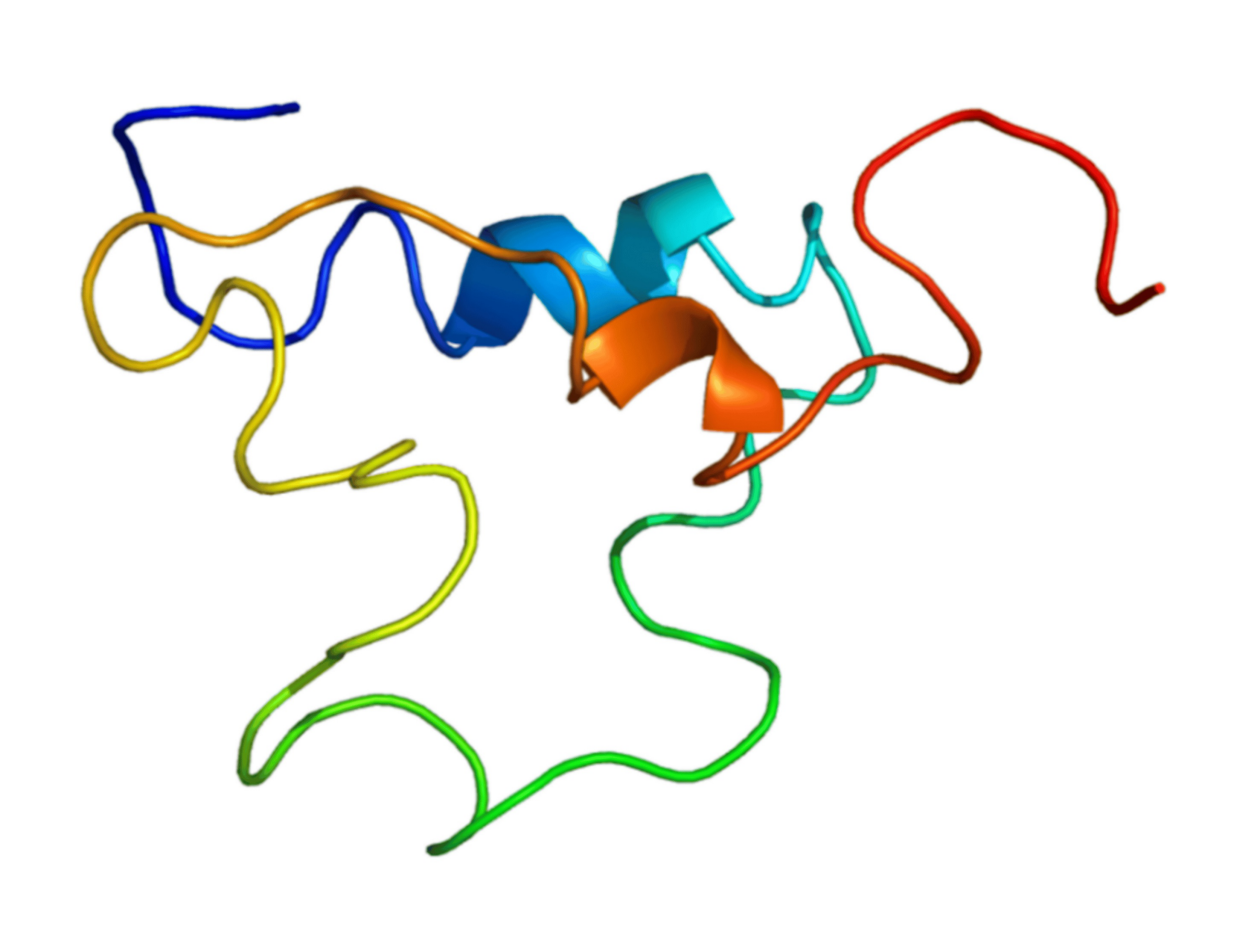
The three-dimensional model of IGF-1 From reference [42]. Copyright 1999-2025 John Wiley & Sons, Inc. Reprinted with permission. IGF-1, insulin-like growth factor 1

Increased milk consumption in adults results in a 10-20% rise in serum IGF-1 levels, and in children, the increase is 20-30% [38,43-46]. Milk consumption also has a pronounced insulinotropic effect, with dairy products raising serum IGF-1 levels more significantly than other protein sources like meat [43]. This observation is supported by several cross-sectional research studies consistently showing that milk consumption is linked to elevated levels of IGF-1 [47-51]. Esterle et al. [38] found that girls consuming over 260 mL of milk daily have significantly higher IGF-1 levels compared to those consuming less than 55 mL. Similarly, a European study involving 2,109 women found a strong positive association between milk consumption and serum IGF-1 levels [52].

Milk and dairy consumption during early pregnancy has been associated with elevated maternal serum IGF-1 levels, potentially overstimulating placental growth and increasing glucose transfer to the fetus [53]. Additionally, milk-induced hyperinsulinemia may exacerbate the physiological IR of pregnancy, thereby raising maternal glucose availability for the fetus [54]. A larger placenta may also produce more placental growth hormone (PGH), a key regulator of fetal IGF-1 synthesis and growth [55]. These factors suggest that milk consumption during pregnancy could contribute to increased fetal growth and higher birth weight [56]. The same study also suggests that the prevalence of fetal macrosomia (birth weight over 4000 g) is increasing in industrialized countries, with rates between 8% and 10%. There is a significant correlation between IGF-1 levels in umbilical cord blood and maternal serum. Studies have shown that IGF-1 and insulin levels are higher in the umbilical cord blood of macrosomic infants compared to those with normal birth weights [57,58]. Maternal IGF-1 enhances the functional capacity of the placenta throughout pregnancy, and both IGF-1 and IGF-2 are crucial for placental and fetal growth. Thus, elevated maternal IGF-1 and insulin levels from milk consumption may contribute significantly to the development of fetal macrosomia.

The GH-IGF-1 axis is closely linked to feeding in newborns. There is also concern that milk intake during pregnancy could negatively impact the early fetal programming of the IGF-1 axis, potentially influencing long-term health risks [40]. Melnik [56] suggests that the IGF-1 axis is programmed early in life, particularly within the first few months. During early pregnancy, maternal IGF-1 influences the placenta, enhancing its functional capacity throughout gestation. IGFs are essential for fetal and placental growth throughout pregnancy. Increased maternal milk consumption during pregnancy can boost nutrient supply to the fetus by enlarging the placenta. In guinea pigs, administering IGF-1 early in pregnancy increased the placental transport of glucose and amino acids, as well as placental and fetal weights. Interestingly, Larnkjaer et al. [59] found an inverse relationship between IGF-1 levels at nine months and at 17 years: a 1 ng/mL increase in IGF-1 at nine months was associated with a 0.95 ng/mL decrease at 17 years. Breastfed infants at two months had an IGF-1 level of 93.3 ± 23.6 ng/mL, while formula-fed infants had higher levels of 129.8 ± 39.8 ng/mL. By age 17, breastfed adolescents had an IGF-1 level of 328 ± 78.5 ng/mL, compared to 292.9 ± 95.0 ng/mL in those not breastfed. These findings support the idea that the IGF-1 axis is programmed early in life by diet [59]. The same study also suggests that newborns who are breastfed exhibit lower serum levels of IGF-1 compared to those fed with formula containing cow’s milk. This indicates that the IGF-1 axis in humans naturally functions at lower levels, and exposure to cow’s milk during pregnancy and the postnatal period may lead to a pathophysiological increase in this axis. This perspective is reinforced by a study comparing 43 breastfed and 43 formula-fed one-week-old term infants. The results showed that postprandial insulin levels were higher in the formula-fed group compared to the breastfed group [60]. Additionally, at two months of age, breastfed infants demonstrated 28.1% lower IGF-1 serum levels compared to the formula-fed infants [59]. Therefore, humans use an inappropriate feeding system that is not ideally suited for their offspring [56]. Maternal consumption of cow’s milk during pregnancy and the use of cow’s milk-based formula for infant feeding may disrupt the proper programming of the insulin/IGF-1 axis during both fetal development and postnatal life [56].

Given that milk protein consumption during pregnancy is linked to higher serum IGF-1 levels and postprandial hyperinsulinemia, it is likely that milk consumption shifts the IGF-1 axis to higher levels early in pregnancy. One of the initial effects of milk consumption during early pregnancy may be an increase in maternal IGF-1 levels, which programs the placenta for enhanced functional capacity, potentially increasing the risk of fetal macrosomia and other IGF-1-related conditions, such as IR, diabetes mellitus, and PCOS. In addition, disruption in the IGF-1 axis during fetal development, resulting in fetal macrosomia, may create a metabolic predisposition to obesity [40,56], which can lead to IR and further increased risk for endocrine disorders.

A prospective study [61] was conducted, in which a total of 3405 mothers completed a 293-item semiquantitative food-frequency questionnaire to obtain information about dairy consumption during the first trimester of pregnancy. Fetal head circumference, femur length, and weight were estimated in the second and third trimesters by ultrasonography [61]. Results of the study showed that pregnant women who consumed more than three glasses of milk daily had babies with higher birth weights, with an average increase of 88 g compared to those who drank zero to one glass per day. Higher milk intake was also associated with larger head circumference, though it didn’t impact birth length. Protein in milk, rather than fat or carbohydrates, seemed to drive this effect, primarily enhancing fetal growth in the third trimester. Similar findings in studies from Denmark, Sweden, India, Canada, and Australia also connect maternal milk intake with higher birth weights, especially due to protein from milk as opposed to other dairy foods or cheese [62-65]. Potential mechanisms may involve increased levels of IGF-I and calcium linked to milk consumption, although further research is needed to explore these pathways.

A cohort study investigated the link between milk consumption during pregnancy and infant birth size using data from 50,117 mother-infant pairs in the Danish National Birth Cohort [62]. Results indicated that higher milk intake was associated with an increase in birth weight, larger abdominal circumference, greater placental weight, and larger head and birth length. Women who drank greater than or equal to six glasses of milk per day had a 49% lower risk of small-for-gestational-age (SGA) births and a 59% higher risk of large-for-gestational-age (LGA) births compared to non-milk drinkers. The increase in birth weight was related to protein intake from milk, not fat. Additionally, in a prospective study of Canadian women [65], which included a high proportion of women who restricted milk intake, low milk consumption was generally linked to a lower average birth weight, though it showed no association with birth length or head circumference.

Intriguingly, with the inverse relation between IGF-1 levels during the first months of life and IGF-1 levels in adulthood observed [59], reduced IGF-1 levels during the postnatal period are linked to elevated IGF-1 levels during adolescence [56]. SGA newborns are reported to have reduced levels of IGF-1 [57]. Girls with low birth weight are at increased risk of early puberty and menstruation, which may progress to conditions such as anovulation, hyperinsulinemic hyperandrogenism, and PCOS [66]. In addition, the IGF-1 axis in SGA or low birth weight newborns seems to shift toward elevated IGF-1 levels over time, potentially contributing to the onset of precocious pubarche and PCOS [56]. Girls with low birth weight and precocious pubarche exhibit elevated IGF-1 levels and IR, leading to rapid progression of puberty and increasing the likelihood of developing PCOS [56].

Factors leading to/exacerbating IR

PCOS is believed to stem from IR or poor SI, which can develop from many factors. Insulin is essential for maintaining glucose homeostasis. Although SI is crucial for metabolic health, the exact mechanisms behind IR are not fully understood. Branched-chain amino acids (BCAAs) are essential amino acids that act as both direct and indirect nutrient signals. Recent research highlighting significant associations between BCAAs and related metabolites with metabolic disease onset, progression, and treatment outcomes suggests a potential causal link between these metabolites and metabolic disease development [67]. While BCAA-rich diets can have benefits, high levels of BCAAs are associated with activation of mammalian target of rapamycin complex 1 (mTORC1), a nutrient-sensing complex; this activation, through IRS-1 serine phosphorylation (Figure 3), may contribute to IR and related metabolic disorders and future IR, including T2DM [68].

**Figure 4:**
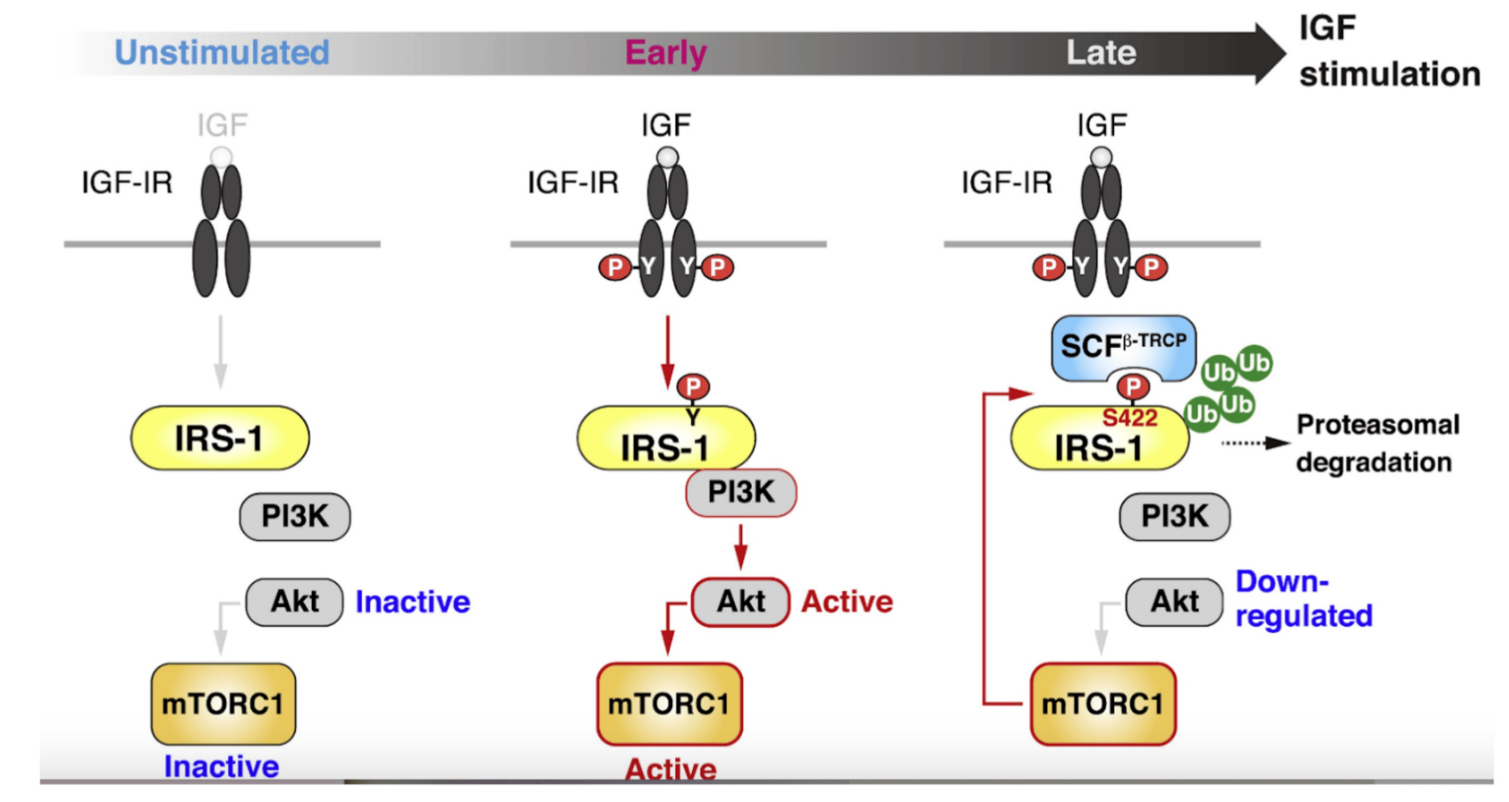
Inhibition of Ser 422 phosphorylation-dependent IRS-1 degradation promotes sustained Akt activation in IGF-stimulated cells From reference [69]. Copyright 2025 Elsevier Inc. Reprinted with permission. Ser 422, serine residue at position 422; IRS-1, insulin receptor substrate-1; Akt, protein kinase B; IGF, insulin-like growth factor

This effect is thought to be a result of leucine, a BCAA. According to Asghari et al. [70], research on the therapeutic effects of BCAA supplementation, particularly leucine, has produced conflicting findings. While some studies suggest leucine improves glucose metabolism and reduces IR in specific models, others indicate that high BCAA intake, especially with a high-protein diet, exacerbates IR by interfering with insulin signaling. This may involve activation of the mTORC1 signaling pathway, which inhibits glucose transport and increases IR through serine phosphorylation of IRS-1. Leucine appears to play a central role in activating mTORC1 and insulin signaling pathways. Additionally, BCAA metabolism may produce mitotoxic metabolites that impair beta-cell function, contributing to IR [70]. Moreover, elevated plasma amino acid levels directly enhance gluconeogenesis and glucose production in healthy individuals [71]. However, this does not impact blood glucose levels due to the concurrent stimulation of insulin secretion induced by amino acids [71], which offsets their direct effects on glucose production [72]. In addition, some studies have indicated that increased intake of BCAAs from meat and dairy products during pregnancy is associated with a higher risk of developing gestational diabetes [2].

Milk proteins are rich in BCAAs and glutamine and, particularly because of their elevated levels of BCAAs, promote greater insulin secretion compared to other proteins, which may influence IR [2]. Dietary proteins are known to stimulate insulin secretion, promoting glucose clearance by peripheral tissues [73]. Whey protein, in particular, has been shown to cause a significant postprandial increase in insulin levels compared to other protein sources like casein or soy, with effects lasting up to 330 minutes [73]. It was found in a study [73] that in healthy individuals, a high-protein meal (50% protein) significantly reduced peak blood glucose levels compared to a carbohydrate-rich meal, with a pronounced insulin response observed after whey consumption. In addition, it was found that while the insulin-stimulating effect of protein may be beneficial for individuals with IR, prolonged hyperinsulinemia in healthy individuals could decrease SI over time, highlighting potential long-term risks. While dairy components such as calcium, magnesium, and probiotics could enhance SI, research results have been inconsistent [2]. Interestingly, according to a study by Skurk et al. [74], BCAA levels may better indicate prediabetic SI impairments than glucose levels, as plasma amino acids respond more persistently to insulin secretion. Elevated BCAA levels could also result from IR due to reduced branched-chain a-ketoacid dehydrogenase (BCKD) enzyme activity, which is inhibited by insulin and elevated free fatty acids [74].

**Figure 5:**
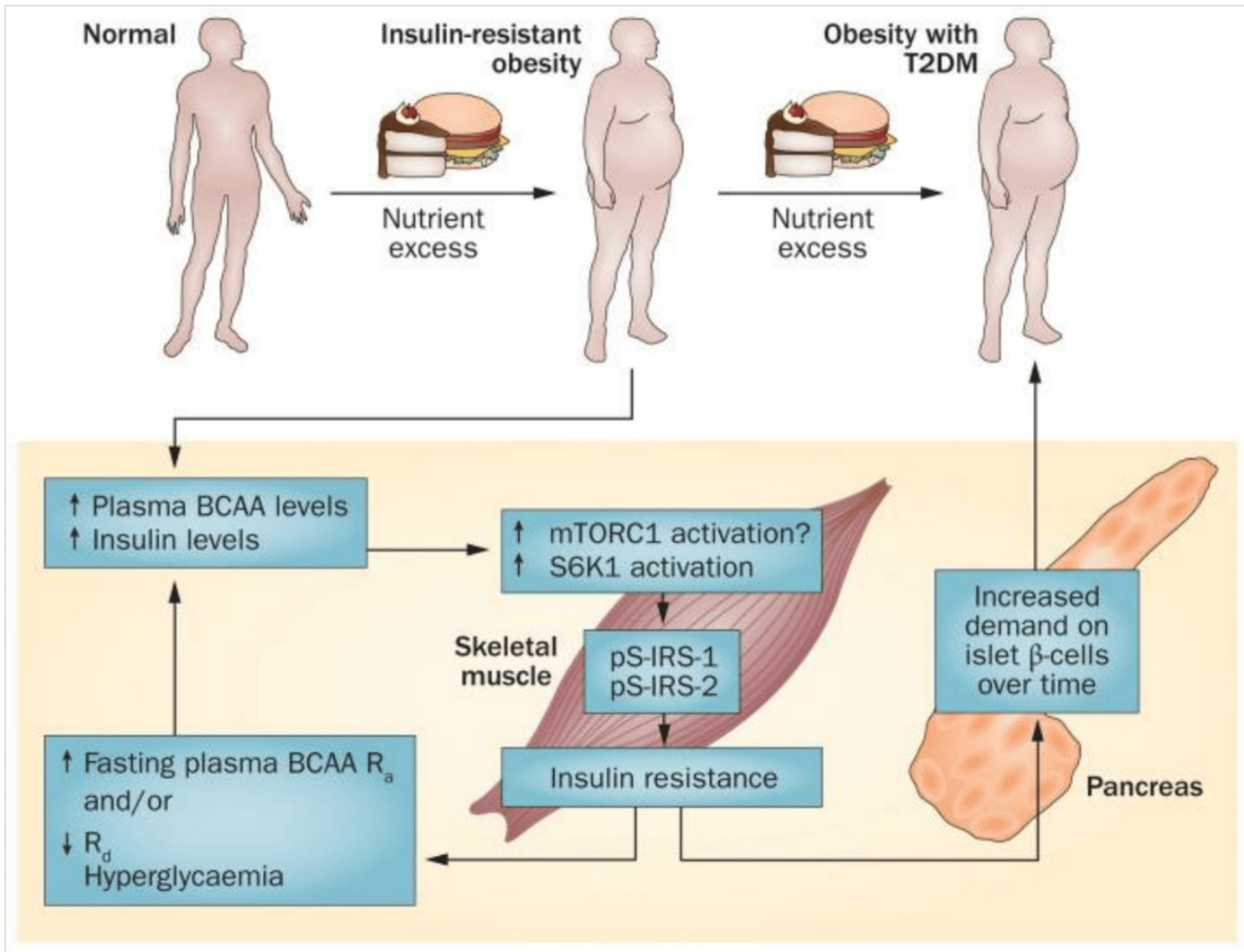
Persistent activation of mTORC1 linking increased plasma BCAA levels to insulin resistance model From reference [75]. Copyright 2014 Macmillan Publishers Limited. Reprinted with permission. mTORC1, mammalian target of rapamycin complex 1; BCAA, branched-chain amino acids

BCAAs, crucial for muscle protein synthesis mediated by mTORC1, need to be paired with mechanical stimuli, such as exercise, to positively impact muscle health [9]. Without these stimuli, high levels of BCAAs could result in negative metabolic effects. Additionally, impaired BCAA metabolism, which can occur in obesity, may lead to further accumulation of BCAAs and their toxic intermediates. These effects are shown in the models in Figures 4, 5.

**Figure 6:**
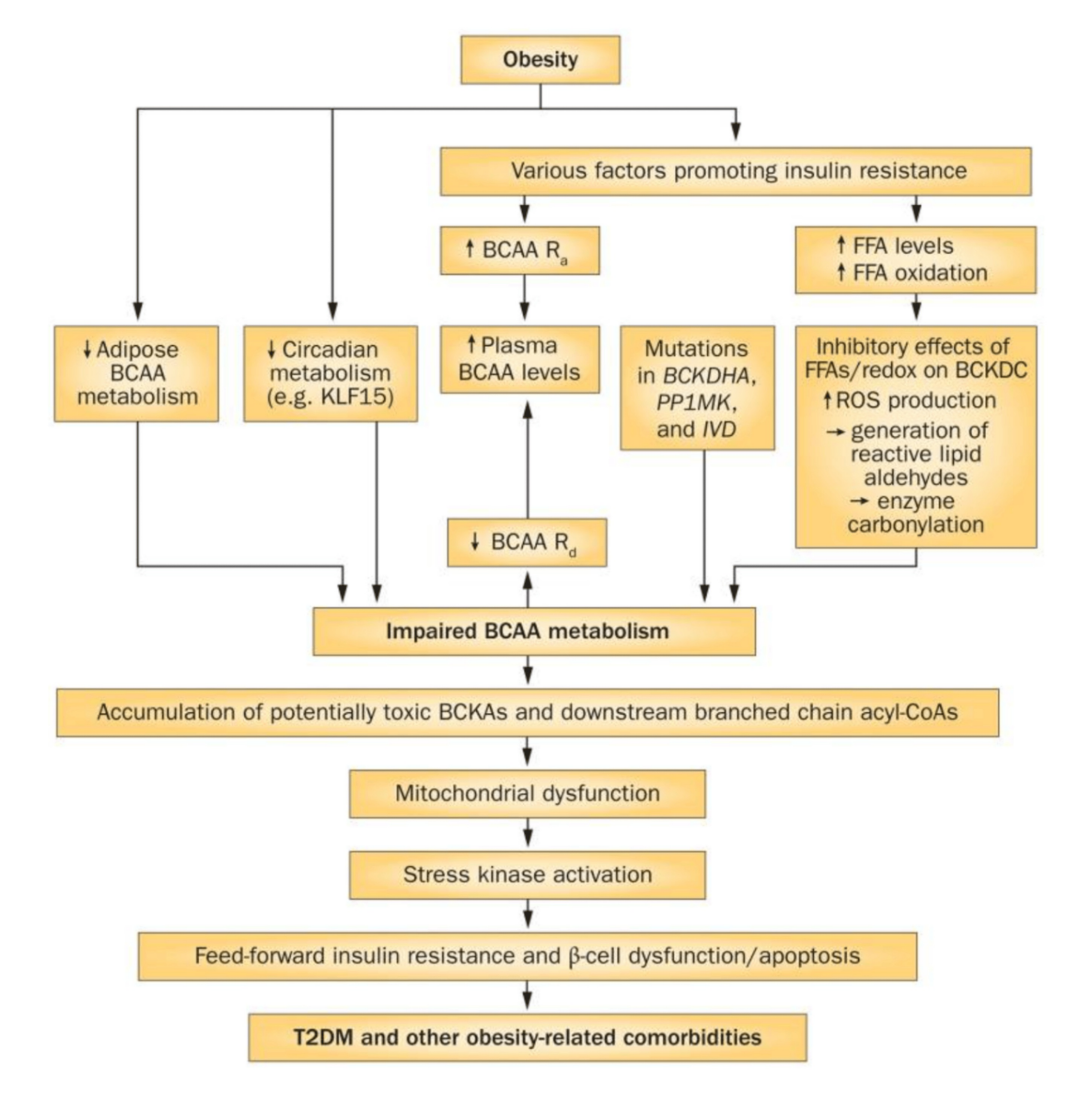
Schematic of how BCAA dysmetabolism links elevated plasma levels of BCAAs and FFAs to T2DM and obesity-related comorbidities From reference [75]. Copyright 2014 Macmillan Publishers Limited. Reprinted with permission. BCAA, branched-chain amino acids; FFAs, free fatty acids; T2DM, type-2 diabetes mellitus

The consumption of milk proteins can lead to increased postprandial insulin levels in obese individuals, contribute to weight gain in overweight adolescents, and potentially exacerbate existing metabolic issues in those who are obese and insulin resistant [9]. Additionally, the consumption of milk proteins, especially whey, can lead to postprandial hyperinsulinemia, which is particularly pronounced in obese individuals. Research comparing fasting insulin levels in lean and obese women with PCOS revealed that β-cell function is increased in both groups; however, obese women exhibited a lower disposition index [37]. In obese women, the reduced fasting state-derived disposition index indicates impaired β-cell compensation relative to IR. The disposition index is a measure that evaluates β-cell function in the context of SI; a lower value signifies that β-cells cannot adequately compensate for the level of IR present. This impairment increases the risk of progressing to IR or type 2 diabetes. This chronic hyperinsulinemia may contribute to β-cell stress and potentially accelerate β-cell apoptosis [9]. Therefore, high dairy consumption, through its stimulation of insulin secretion, ultimately contributes to the development of IR. This finding can be supported by a recent study involving 500 obese participants from the Weight Loss Maintenance (WLM) trial [76], where blood samples collected at baseline and after a six-month behavioral and dietary intervention (DASH diet) were examined. Results showed that weight loss during the intervention had a weak correlation with improvements in the HOMA score. Interestingly, targeted metabolic profiling of baseline plasma samples revealed that the BCAA-related principal component factor score was a strong predictor of enhanced SI following the intervention, while lipid-related factors (e.g., long-chain fatty acids and ketone metabolites and medium-chain acylcarnitines) showed no predictive association [77].

However, recent research raises questions about whether physiological increases in BCAAs are sufficient or necessary to cause these conditions. Studies in rat models suggest that elevated BCAA levels alone may not directly induce IR or T2DM [68]. Lynch and Adams [75] suggest two potential mechanisms that are likely to explain the association between elevated levels of BCAAs and metabolic diseases: one proposed mechanism links elevated BCAAs to metabolic disease through persistent activation of mTORC1, a nutrient-sensing complex. However, evidence suggests that mTORC1 activation alone is neither necessary nor sufficient to induce IR. The alternative mechanism, a BCAA dysmetabolism model, suggests that individuals with the highest plasma BCAA levels (often linked to metabolic dysfunction) may have impaired BCAA breakdown. This impairment leads to the buildup of BCAA metabolites, which could drive mitochondrial dysfunction, activate stress kinases, and cause β-cell apoptosis, all of which are associated with IR and type 2 diabetes. Elevated BCAA levels may serve as markers of this metabolic imbalance rather than direct causes. They concluded that while various mechanisms have been suggested to explain the potential link between elevated BCAA levels and the development of IR or obesity, it is more plausible that increased BCAA levels serve as indicators of diminished SI rather than being direct causes.

Conversely, Lee et al. [78] investigated the associations of BCAAs with SI, acute insulin response (AIR), and metabolic clearance of insulin (MCRI) in a multiethnic cohort of 685 participants without diabetes of the IR (290 Caucasians, 165 African Americans, and 230 Hispanics). The results showed that elevated plasma BCAAs were inversely associated with SI and MCRI and positively associated with fasting insulin. They concluded that elevated plasma BCAA levels are linked to the onset of diabetes and related metabolic dysfunctions. 

Estrogen/estrone in milk

While dairy products are widely recognized as a nutritious food source, they are also known for containing hormones that can influence physiological and pathological processes [79]. The potential for these hormones to disrupt normal endocrine system functions has raised widespread concern globally [80]. Recent studies have raised concerns about its safety due to the presence of steroid hormones, such as estrogens [81-83], especially when derived from pregnant cows. Dairy cows are usually milked until the seventh or eighth month of pregnancy, a period when the estrogen content in their milk increases due to hormone production by the placenta [84]. During the third trimester, estrogen levels in milk can be as much as 20 times higher than in milk from non-pregnant cows, with estrone (E1) and 17β-estradiol (E2) levels ranging from 25 to 118 pg/mL, which is almost as high as a pre-ovulatory woman [84]. Estrogens, including estrone (E1), estradiol (E2), and estriol, are hormones that can act through nuclear receptor pathways and other signaling mechanisms and play a role in regulating reproduction [85]. Although estrogens are naturally occurring hormones, higher levels of estrogen have been linked to an increased risk for reproductive and endocrine disorders, raising questions about the possible effects on human and animal health. Some human studies have shown hormonal changes, such as increases in E1 and progesterone levels after milk consumption. Meanwhile, animal studies have reported varying effects on reproductive systems [84]. While some studies have linked milk consumption to reproductive issues, the evidence remains mixed, with some suggesting no risk and others indicating protective effects. This raises a critical question: Could the estrogens in milk impact hormone levels and reproductive health?

Dairy products, such as milk, are notable sources of estrogen in the diet, with some research indicating that the estrogen content in milk may be associated with reproductive health concerns [85]. Research indicates that commercial milk, sourced from both pregnant and non-pregnant animals, contains significant levels of estrogens, progesterone, and other placental hormones, which ultimately enter the human food chain [86]. It is estimated that dairy consumption accounts for 60-80% of dietary estrogen intake [87]. These hormones persist through processing and are present in both raw whole cow’s milk [85,88] and commercial dairy products [89]. Their concentration increases with higher milk fat content, with no discernible difference between organic and conventional dairy products [85]. Once ingested, these bovine hormones are metabolized into estrone and estradiol in the human body, potentially influencing reproductive health [90]. Human studies have shown links between dairy intake and changes in plasma steroid hormone levels [91] as well as gonadotropin secretion [86]. This suggests that bovine-derived steroids absorbed from dairy products could potentially impact the hypothalamic-pituitary-gonadal axis or directly affect oocytes and their intra-ovarian or intra-follicular environments [92]. These effects may occur through mechanisms such as altered gene expression and disrupted neuroendocrine signaling [92].

Grgurevic et al. [84] explored the potential impact of estrogen from cow’s milk on mice. During the third trimester of pregnancy, cows produce milk with significantly higher estrogen levels, up to 20 times greater than milk from non-pregnant cows. For the experiment, 30 male and 30 ovariectomized female mice were given varying doses of milk with different estrogen concentrations over an eight-day period. The experimental groups received milk with either natural estrogen levels or added estrogen at concentrations of 10 and 100 ng/mL, while a control group received no milk. The findings showed that milk with natural estrogen or the lower dose (10 ng/mL) did not alter the mice’s hormone levels or reproductive organs. However, the group that received the highest dose (100 ng/mL) had higher estrogen levels, increased uterine weight in females, and decreased testosterone levels in males. The researchers concluded that such high estrogen levels are much greater than those typically found in cow’s milk, suggesting that these effects would not occur under normal conditions [84].

Maruyama et al. [86] emphasize that cow’s milk, particularly from pregnant cows, serves as a significant source of exogenous hormones, including estrone (E1), estradiol (E2), and progesterone. Research indicates that these hormones can be absorbed by the human body through milk consumption, potentially altering hormone levels. The study investigated how cow’s milk consumption impacts hormone levels in a group of women (five participants with regular menstrual cycles) who drank 500 mL of milk nightly for 21 days during the second of three menstrual cycles, with basal body temperature and ovulation timing monitored to assess any effects. Hormone levels were measured using advanced immunoassays. It was found that ovulation timing remained unaffected by milk intake in four of the five participants, but one participant experienced delayed ovulation, which occurred after discontinuing milk consumption. Overall, milk intake was associated with significant hormonal changes. In addition, it was determined that epidemiological trends point to earlier puberty in girls, which may be associated with higher body mass index (BMI) and exposure to external estrogens like those in milk. While normal menstrual cycles in women appear largely unaffected, conditions such as oligomenorrhea could experience delayed ovulation with excessive milk intake. Overall, the findings suggest that elevated hormone levels in milk could affect ovulation timing and overall reproductive health. However, challenges remain in accurately measuring estrogen levels in milk due to inconsistencies in reported data [86].

In contrast, Wolford and Argoudelis [93] found no evidence that the estrogens present in milk have any biological effects on humans. The study investigated the estrogen levels in both raw and commercially processed dairy products, finding considerable variation among cows, likely influenced by changes in hormonal levels during the estrus cycle. Estrone (E1) concentrations were higher than estradiol (E2-17β) in both milk and butter, with these estrogens being distributed across the fat and non-fat components of the milk. Tracer studies indicated that about 80% of E1 was linked to the fat content, whereas 65% of E2-17β was similarly distributed. Further protein binding analysis showed that most estrogens in milk were bound to proteins, especially whey proteins. Butter contained the highest estrogen levels, although these amounts were too minimal to trigger biological effects. Overall, while milk and dairy products contain estrogens, their concentrations are too low to have a biological impact, and additional research is needed to investigate the potential presence of estrogen conjugates [93].

This result is corroborated in a study [85] that involved two experiments aimed at testing how pasteurization and homogenization (P-H) treatments and fat content in milk affect the concentrations of estrone (E1) and 17β-estradiol (E2). In this study, estrone (E1) and 17β-estradiol (E2) were consistently found in milk and dairy products, with E1 levels being significantly higher than E2. A small but statistically significant difference in E2 levels was found between conventional milk and organic milk, with organic varieties having slightly higher concentrations; however, this difference was not of biological significance. The estrogen levels found in dairy products are much lower than the amounts naturally produced by humans, with women generating thousands of times more E1 and E2 daily than are present in dairy products. It was concluded that the concentrations of these estrogens were very low compared to other research, potentially due to variations in the analytical methods used, but the P-H treatment did not significantly affect the levels of E2 in milk, making any potential health impacts from dairy-derived estrogens improbable [85].

## Conclusions

Milk consumption has complex effects on reproductive and metabolic health, particularly due to its protein content and hormone levels. Milk protein, rich in BCAAs, can influence SI by increasing insulin levels, which may be problematic for individuals with IR, obesity, or conditions like PCOS. Elevated insulin levels can exacerbate metabolic issues, making blood sugar regulation more difficult. Additionally, the estrogen content in milk, especially from pregnant cows, raises concerns about its potential impact on hormone balance. Estrogen levels in dairy products can be significantly higher during late pregnancy, and while the overall concentration in milk is lower than natural human estrogen production, prolonged exposure could contribute to hormonal fluctuations. Some studies suggest that these hormones may affect menstrual cycles, ovulation, and fetal development, particularly by influencing maternal SI and increasing the risk of fetal macrosomia in pregnant individuals with gestational diabetes or IR. However, evidence remains inconclusive, with some studies finding minimal impact on reproductive health.

The broader implications of milk consumption on reproductive health remain an area of active research, as findings on its hormonal effects have been mixed. While some studies suggest milk intake could alter ovulation timing or contribute to subtle hormonal shifts, others find no significant effects on menstrual cycles or overall reproductive function. Additionally, factors such as milk fat content and processing methods can influence hormone concentrations, though their biological effects in humans remain uncertain. It is important to approach milk consumption in moderation, especially for individuals who are already at risk for metabolic disorders or reproductive health issues. Until more research is available, it may be wise to pay attention to the source and type of dairy consumed and be mindful of any health changes that could be linked to milk intake.
